# HOMA-IR is associated with significant angiographic coronary artery disease in non-diabetic, non-obese individuals: a cross-sectional study

**DOI:** 10.1186/s13098-015-0085-5

**Published:** 2015-11-14

**Authors:** Márcio Mossmann, Marco V. Wainstein, Sandro C. Gonçalves, Rodrigo V. Wainstein, Gabriela L. Gravina, Marlei Sangalli, Francine Veadrigo, Roselene Matte, Rejane Reich, Fernanda G. Costa, Marcello C. Bertoluci

**Affiliations:** Cardiology Division, Hospital de Clínicas de Porto Alegre, Universidade Federal do Rio Grande do Sul, Ramiro Barcellos 2350, Porto Alegre, 90035-903 Brazil; Universidade Federal do Rio Grande do Sul, Ramiro Barcellos 2350, Porto Alegre, 90035-903 Brazil; Internal Medicine Department, Hospital de Clínicas de Porto Alegre and Programa de Pós-Graduação em Medicina: Ciências Médicas, Universidade Federal do Rio Grande do Sul, Ramiro Barcellos 2350, Room 700, Porto Alegre, 90035-903 Brazil

## Abstract

Insulin resistance is a major component of metabolic syndrome, type 2 Diabetes Mellitus (T2DM) and coronary artery disease (CAD). Although important in T2DM, its role as a predictor of CAD in non-diabetic patients is less studied. In the present study, we aimed to evaluate the association of HOMA-IR with significant CAD, determined by coronary angiography in non-obese, non-T2DM patients. We also evaluate the association between 3 oral glucose tolerance test (OGTT) based insulin sensitivity indexes (Matsuda, STUMVOLL-ISI and OGIS) and CAD. We conducted a cross-sectional study with 54 non-obese, non-diabetic individuals referred for coronary angiography due to suspected CAD. CAD was classified as the “anatomic burden score” corresponding to any stenosis equal or larger than 50 % in diameter on the coronary distribution. Patients without lesions were included in No-CAD group. Patients with at least 1 lesion were included in the CAD group. A 75 g oral glucose tolerance test (OGTT) with measurements of plasma glucose and serum insulin at 0, 30, 60, 90 and 120 min was obtained to calculate insulin sensitivity parameters. HOMA-IR results were ranked and patients were also categorized into insulin resistant (IR) or non-insulin resistant (NIR) if they were respectively above or below the 75th percentile (HOMA-IR > 4.21). The insulin sensitivity tests results were also divided into IR and NIR, respectively below and above each 25th percentile. Chi square was used to study association. Poisson Regression Model was used to compare prevalence ratios between categorized CAD and IR groups. Results: Fifty-four patients were included in the study. There were 26 patients (48 %) with significant CAD. The presence of clinically significant CAD was significant associated with HOMA-IR above p75 (Chi square 4.103, p = 0.0428) and 71 % of patients with HOMA-IR above p75 had significant CAD. Subjects with CAD had increased prevalence ratio of HOMA-IR above p75 compared to subjects without CAD (PR 1.78; 95 % CI 1.079–2.95; p = 0.024). Matsuda index, Stumvoll-ISI and OGIS index were not associated with significant CAD. We concluded that, in patients without diabetes or obesity, in whom a coronary angiography study is indicated, a single determination of HOMA-IR above 4.21 indicates increased risk for clinical significant coronary disease. The same association was not seen with insulin sensitivity indexes such as Matsuda, Stunvoll-ISI or OGIS. These findings support the need for further longitudinal research using HOMA-IR as a predictor of cardiovascular disease.

## Background

Insulin resistance (IR) is a major component of several significant clinical conditions including metabolic syndrome, type 2 diabetes (T2DM) and cardiovascular disease [1, 2]. In T2DM patients, IR is associated with endothelial dysfunction, a pro-inflammatory state and cardiovascular disease, being a central mechanism promoting atherosclerosis [3]. The impact of IR in atherogenesis is classically attributed to the effects of hyperinsulinemia and hyperglycemia, both factors being difficult to be understood separately as predictors of coronary atherosclerosis. However, the association between IR and coronary artery disease may be independent from traditional risk factors [4]. It has been described that, in healthy asymptomatic adults aged 60–72 years, high insulin levels can predict progression of coronary artery calcification as seen by coronary artery calcium score after 2 years. This was shown to be independent from risk factors such race, dyslipidemia, hypertension and diabetes, being a strong indicator that IR is an independent predictor of coronary artery progression [5].

There is great need of identification of practical surrogate markers of IR which could be useful for coronary risk stratification in patients at risk for coronary artery disease (CAD). IR has significant biological plausibility and some markers have been evaluated with variable results. In a previous study from our group, we observed that HOMA-IR could be an interesting candidate for a coronary disease marker in a mixed population of T2DM and non-diabetic patients submitted to coronary angiography. In that study, we observed an association between HOMA-IR and the presence of sub-clinical coronary atherosclerosis, although the results may have been impacted by the presence of a high number of T2DM patients and anti-hyperglycemic treatment [6].

In the present study our primary aim was to evaluate the association of HOMA-IR, a fasting IR marker, with significant coronary artery disease, defined by a coronary burden score obtained from coronary angiography in a non-obese, non-T2DM population, without anti-hyperglycemic treatment. We also intended to evaluate the same association with the insulin sensitivity indexes (Matsuda, Stumvoll-ISI and OGIS) which are based on glycemic and insulin determinations after an oral glucose load [7].

## Methods

### Study design and patients

**Fig. 1:**
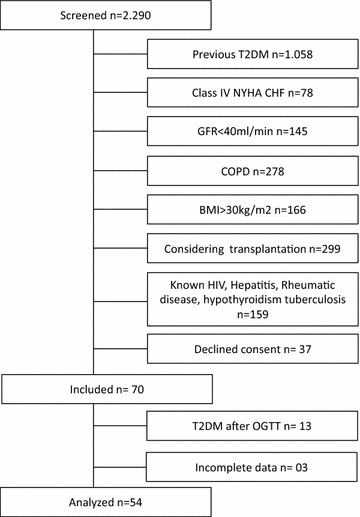
Inclusion of patients-flowchart

We conducted a cross-sectional study with patients referred for coronary angiography at a reference center of cardiology at Hospital de Clínicas of Porto Alegre, Brazil. Between October 2012 and March 2014, a total of 2290 patients referred to coronary angiography for chest pain or myocardial ischemia in non-invasive tests were screened. Fifty-four patients who fulfilled inclusion and exclusion criteria and accepted to participate in the study were included (Fig. 1). All patients signed the written informed consent, and the Hospital Ethics Committee approved the study protocol.

Inclusion criteria were: age between 30–75 years and suspected coronary artery disease. Exclusion criteria were: a known history of diabetes; presence of class IV NYHA congestive heart failure; acute coronary syndrome in the last 30 days; glomerular filtration rate below 45 mL/min/1.73 m^2^; chronic obstructive pulmonary disease; body mass index above 30 kg/m^2^; previous organ transplantation, current evaluation for transplantation; presence of rheumatic, endocrine or infectious chronic diseases such as arthritis, hypothyroidism, HIV infection, hepatitis, tuberculosis. We also excluded patients using any medication that could modify glucose-insulin metabolism such as: insulin, metformin, sulfonylureas and any other oral drug for diabetes. We also excluded patients taking corticosteroids, HIV anti-retrovirals, carbamazepine, phenytoin, drugs for cancer, immunosupressor drugs, nitrofurantoin, anti-malarics, lithium and anti-psycotic drugs.

### Biochemical investigation

In a maximum of 14 days (average 6 days) after the coronary angiography, patients were scheduled to return in 12-h fasting for a 75 g of oral glucose tolerant test (OGTT) with the determination of plasma glucose and serum human insulin at baseline and then at 30, 60, 90 and 120 min after the glucose load. Serum ultra sensitive C-reactive protein, creatinine, lipid profile, and glycated hemoglobin were also measured. Physical examination including anthropometric measurements including height, weight, body mass index and abdominal circumference, was performed at the same time.

### Coronary angiography analysis

Coronary angiographies were performed using the Axiom Artis Siemens equipment (Germany) in all patients. All angiographic measurements were made by two experienced interventional cardiologists who were blinded to insulin resistance status and other clinical variables. Angiographic analyses were made by visual (non-quantitative) estimates of luminal narrowing in at least two different orthogonal projections. Coronary artery disease burden was classified as the “anatomic burden score” created for the analysis of the “coronary anatomy versus ischemia” in the COURAGE trial [8]. Briefly, it consists in a grading scale of 17 progressive degrees of severity starting from zero, which is the complete absence of coronary disease, to 17 which corresponds to trivascular disease including lesions at proximal left anterior descending artery, plus left circumflex artery and right coronary involvement. To meet criteria, each lesion must represent at least 50 % diameter stenosis on the coronary distribution. We defined patients into two groups: Those who failed to meet the 50 % diameter stenosis threshold were defined as “NO CAD” (anatomic burden score = 0) and those with anatomic burden score equal or above one, as CAD.

### Insulin resistance analysis

We used HOMA-IR [9] as a fasting insulin-resistant test and three insulin sensitivity surrogate markers based on OGTT which were validated against the hyperinsulinemic-euglycemic clamp: OGIS, Stumvoll-ISI, and Matsuda index. Each test was calculated as follows:

#### Homeostasis model assessment (HOMA-IR)

HOMA-IR is a model of relationship of glucose and insulin that predicts fasting steady-state glucose and insulin concentrations. The product of fasting glucose and fasting insulin is an index of hepatic insulin resistance, calculated as follows [9]:

$$ {\text{HOMA-IR = [FPI }} \times {\text{FPG]/22}} . 5 $$

where: FPI, fasting plasma insulin (mU/L); FPG, fasting plasma glucose (mmol/L).

#### Matsuda index

This composite whole-body insulin sensitivity index (ISI) is based on post oral glucose load (OGTT) insulin and glucose values in relation to its corresponding fasting values. It is dependent of both hepatic and peripheral tissue sensitivity to insulin and is calculated as follows [10]:

$$ {\text{ISI}}_{\text{MATSUDA}}  { = 10{,}000} / {\text{SQRT [FPG }} \times {\text{FPI }}\left] {\, \times \,} \right[{\text{MG }} \times {\text{MI]}} $$

where: SQRT, square root; FPG, fasting plasma glucose (mg/dl); FPI, fasting plasma insulin (mU/mL); MG, mean glucose (mg/dL); MI, mean insulin.

#### Stumvoll index (ISI)

The Stumvoll index is also derived from OGTT including variables such as insulin at 120 min, glucose at 90 min and Body Mass Index. ISI Stumvoll is highly correlated to hyperinsulinemic-euglycemic clamp in respect to insulin resistance [11].

$$ {\text{ISI}}_{\text{STUMVOLL}}  { = }\left[ { 0. 2 2 6 2} \right]\,{-}\, [ 0. 0 0 3 2 \times {\text{BMI}}\left] {\,{-}\,} \right[ 0. 0 0 0 0 6 4 5 \times {\text{INS}}_{ 1 2 0} \left] {\,{-}\,} \right[ 0. 0 0 3 7 5 \times {\text{GLUC}}_{ 9 0} ] $$

where: BMI, body mass index (kg/m^2^); INS_120_, plasma insulin at 120 min after OGTT (pmol/L); GLUC_90_, plasma glucose at 90 min after OGTT (mg/dL).

#### Oral glucose insulin sensitivity index (OGIS)

OGIS is an index of insulin sensitivity calculated using a model derived principle from the OGTT glucose and insulin concentration, being equivalent to glucose clearance calculated and validated in the hyperinsulinemic-euglycemic clamp in patients with T2DM [12]. It requires fasting, 90 and 120 min of both glucose and insulin concentrations during the OGTT. The calculation of OGIS is published elsewhere [12] and can be calculated at: http://webmet.pd.cnr.it/ogis/index.php.

Results for each insulin resistance marker were ranked and divided into percentiles. As HOMA-IR correlates directly with insulin resistance, we selected p75 as the best cut-off value (HOMA-IR value 4.21). Patients were categorized into insulin resistant (IR) group or Non-Insulin Resistant (NIR) group if they were respectively above and below 4.21. As Matsuda, Stumvoll ISI and OGIS indexes are indicators of insulin sensitivity, we used the p25 cut-off and defined IR and NIR respectively below and above the cut-off value. The results were compared in respect of the presence or absence of coronary artery disease burden score.

### Statistical analysis

Continuous variables with parametric distribution were expressed as mean ± standard deviation, whereas non-parametric variables levels were expressed as median (95 % confidence interval) and analyzed using Mann–Whitney’s test. Categorical data were expressed as frequencies and their differences were analyzed using the Chi square test in the general characteristic Table 1. The association between CAD and HOMA-IR, Matsuda index, Stumvoll index and OGIS index with the presence of CAD was assessed by Chi square and through prevalence ratio obtained from Poisson regression model, which was calculated considering CAD as the independent variable and the insulin resistant markers as the predictors (Table 2). Statistical analyses were performed using SPSS version 15.0 (SPSS Inc., Chicago, IL).

**Table 1:** Characteristics of patients based on HOMA-IR above or below the 75th percentile

	HOMA > p75	HOMA < p75	*p*
N	14	40	
Age (years)	57.78 ± 5.16	58.37 ± 7.29	0.78
Men (%)	50	40	0.51
BMI (kg/m^2^)	29.59 ± 5.23	25.94 ± 4.05	0.0096
AC (cm)	101.69 ± 10.47	90.67 ± 10.36	0.0016
Systolic BP (mmHg)	134.57 ± 22.49	136.25 ± 20.17	0.79
Diastolic BP (mmHg)	78.00 ± 10.65	78.77 ± 13.77	0.96
Serum creatinine (mg/dL)	0.76 ± 0.26	0.75 ± 0.18	0.90
Fasting glucose (mg/dL)	97.35 ± 5.10	91.35 ± 9.22	0.02
2 h OGTT glucose (mg/dL)	127.50 ± 36.54	126.30 ± 26.59	0.89
Fasting insulin	21.07 ± 2.65	8.71 ± 3.37	0.001
2 h OGTT insulin (mUI/L)	180.13 ± 102.06	87.59 ± 58.52	0.002
HOMA-IR	5.06 ± 0.71	1.97 ± 0.81	0.0001
HbA1c (%)	5.67 ± 0.25	5.65 ± 0.35	0.84
Total cholesterol (mg/dL)	190.21 ± 48.01	177.98 ± 48.55	0.42
HDLc (mg/dL)	42.36 ± 11.26	45.82 ± 10.89	0.31
Triglicerides (mg/dL)	168.14 ± 111.35	122.32 ± 76.55	0.094
US CRP (mg/dL)	3.60 ± 3.56	5.36 ± 6.21	0.52
Urinary albumin/creatinine (mg/g)	28.392 ± 41.58	25.93 ± 40.61	0.63
Hypertension (%)	78.5	82.0	0.77
Previous MI (%)	21.4	13.1	0.46
Smokers (%)	21.4	33.3	0.51
Statin use (%)	84.6	63.1	0.15
ACEI (%)	38.4	23.7	0.30
β-blocker (%)	69.2	57.8	0.46
ASA (%)	69.2	60.0	0.57
Nitrates (%)	23.0	15.8	0.56

*AC* abdominal circumpherence, *BMI* body mass index, *OGTT* oral glucose tolerance test, *ASA* acetyl salicilic acid, *US-CRP* ultra sensitive C-reactive protein, *ACEI* angiotensin conversion enzyme inhibitor

**Table 2:** Characteristics of patients divided by the presence of coronary artery disease

	No CAD	CAD	p
N	29	26	
Age (years)	59.8 ± 5.86	56.5 ± 7.28	0.069
Men (%)	31.0 (9/29)	53.8 (14/26)	0.086
BMI (kg/m^2^)	26.6 ± 4.56	27.2 ± 4.7	0.64
AC (cm)	92.46 ± 11.4	94.3 ± 11.2	0.53
Systolic BP (mmHg)	134.5 ± 19.8	134.1 ± 18.2	0.92
Diastolic BP (mmHg)	76.5 ± 13.5	79.2 ± 9.16	0.39
Serum creatinine (mg/dL)	0.70 ± 0.17	0.81 ± 0.21	0.036
Fasting glucose (mg/dL)	93.1 ± 9.1	92.1 ± 8.5	0.672
2 h OGTT (mg/dL)	130.2 ± 26.2	121.3 ± 31.9	0.265
Fasting insulin	10.6 ± 5.0	13.3 ± 7.3	0.28
2 h OGTT insulin (mUI/L)	108.5 ± 92.4	110.5 ± 67.4	0.930
HbA1c (%)	5.69 ± 0.35	5.58 ± 0.29	0.239
Total cholesterol (mg/dL)	180.7 ± 52.1	183.3 ± 44.4	0.844
HDLc (mg/dL)	46.9 ± 11.4	43.5 ± 10.9	0.266
Triglicerides (mg/dL)	128.8 ± 88.3	141.9 ± 88.0	0.58
US CRP (mg/dL)	4.71 ± 5.53	5.03 ± 5.8	0.83
Urinary albumin/creatinine (mg/g)	23.4 ± 40.5	17.8 ± 30.8	0.78
Hypertension (%)	74.2	88.0	0.312
Previous MI (%)	6.8	23.1	0.06
Smokers (%)	35.5	20.0	0.245
Statin use (%)	48.4	91.3	0.001
ACEI (%)	19.4	39.1	0.133
Beta-blocker (%)	45.2	78.3	0.024
ASA (%)	39.4	87.0	0.002
Nitrates (%)	12.9	21.7	0.472

*BMI* body mass index, *Abd Circ* abdominal circumference, *HbA1c* glycosilated haemoglobin, *LDLc* low density lipoprotein cholesterol, *HDLc* high density lipoprotein cholesterol, *US CRP* ultra sensitive C reactive protein, *ACEI* angiotensin conversing enzyme inhibitor, *ASA* acetylsalicylic acid; urinary albumin/creatinine, insulin and HOMA-IR were compared through Mann–Whitney Rank test. All other continuous variables were tested through Student t test. Categorical variables were tested through Chi square

## Results

The flowchart of the sampling process is depicted in Fig. 1. Clinical and biochemical characteristics of included patients are shown in Tables 1 and 2. Fifty-four patients were analyzed and 26 (48 %) had significant CAD.

**Fig. 2:**
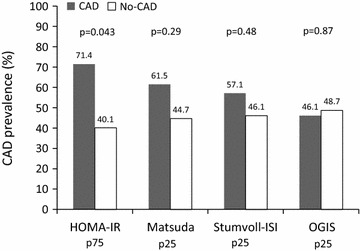
Prevalence of coronary artery disease in relation to insulin resistance parameters

When groups were divided above and below the 75th percentile of HOMA-IR (Table 1) they were similar in age, gender, systolic and diastolic blood pressure, HbA1c, serum creatinine, urinary albumin/creatinine ratio, lipid profile, presence of hypertension and smokers. They were also similar in respect of the use of statins, aspirin, betablockers and anti-hypertensive drugs. The group HOMA above p75, as expected, had increased BMI, abdominal circumference, fasting and 2 h post-OGTT insulin levels as well as fasting glucose levels, although in the normal range. The presence of CAD was significantly associated with HOMA-IR above p75 (Chi square 4.103, p = 0.0428). In patients with HOMA-IR above the 75th percentile 71.4 % had significant CAD (predictive positive value) (p = 0.048) while in the group with HOMA-IR below p75, 40 % had CAD (Fig. 2).

When groups were divided by the presence or not of significant CAD (Table 2), they were similar in respect of age, BMI, abdominal circumference, systolic and diastolic blood pressure, smokers, fasting plasma glucose and 2 h post-OGTT plasma glucose, glycated hemoglobin and serum insulin. There was a non-significant trend for increased number of men in the CAD group and serum creatinine was increased in the CAD group (p = 0.036), although values were within the normal range. As expected, the number of statin, beta-blocker and aspirin users were higher in CAD group. Subjects with CAD had increased prevalence ratio of HOMA-IR above p75 compared to subjects without CAD (PR 1.78; 95 % CI 1.079–2.95; p = 0.024) (Table 3).

**Table 3:** Prevalence ratio for HOMA-IR, Matsuda Index, Stumvoll-ISI and OGIS index in patients with and without coronary artery disease (CAD)

	Cut off	Percentile	PR	95 % CI	PPV-CAD (%)	p
HOMA-IR	4.21	75	1.78	(1.079–2.955)	71.4	0.024
Matsuda	77.06	25	1.37	(0.789–2.399)	61.5	ns
OGIS	304.5	25	0.94	(0.485–1.851)	46.1	ns
Stumvoll ISI	4.39	25	0.76	(0.362–1.635)	38.4	ns

*PR* prevalence ratio, *CI* confidence interval, *PPV* positive predictive value for CAD, *HOMA-IR* homeostases model assessment-insulin resistance, *ISI* insulin sensitive index, *ns* non significant

The insulin sensitivity indexes Matsuda, Stumvoll-ISI and OGIS were not associated with significant CAD (Chi square: p = 0.29, 0.48 and 0.87, respectively). There was no significance in prevalence ratio for CAD when Matsuda, Stunvoll-ISI and OGIS were considered above and below each p25, respectively: PR 1.37, p = ns; PR 0.76, p = ns and PR 0.94, p = ns.

## Discussion

In this study, we found a significant association between IR represented by HOMA-IR above p75 and the presence of significant CAD in non-diabetic, non-obese patients referred for coronary angiography. A measurement of HOMA-IR above 4.21 had a positive predictive value of 71.4 % for the presence of 50 % or greater stenosis in at least one coronary artery. The same association was not found when using the insulin sensitivity tests derived from the oral glucose tolerance tests such as Matsuda, Stumvoll ISI and OGIS below the respective p25 cut-off.

These findings are in accordance with our previous study [6] in which HOMA-IR was associated to CAD, although in a population of both T2DM and non-DM patients. In that study, HOMA-IR above 6.0 was predictive for significant CAD (PPV 82.3 %). If cut-off value is reduced to 4.21, PPV would fall to 69.4 %. Thus, at the same cut off level, in the present study, HOMA-IR may be more predictive of CAD in non-diabetic patients. We presume that in part, this finding could be due to the more homogeneous population in the present study. The main point is that the present results reinforce the impact of HOMA-IR as an important cardiovascular risk predictor in non-diabetic population.

Few studies have previously evaluated HOMA-IR as a cardiovascular risk factor. Srinivasan et al. studied 61 T2DM who were submitted to coronary arteriography in a cross-sectional study. The log-HOMA-IR was positively associated with the severity of coronary risk [13]. Similar results were obtained in the San Antonio Heart Study which found a significant association between HOMA-IR and risk of CVD after adjustment for multiple covariates [14]. In a large observational study, Hedblad B et al. [15] studied normoglycemic individuals without previous cardiovascular events who were divided according to the presence or not of insulin-resistance on the basis of the 75th percentile of HOMA-IR and followed for 6 years. They found that individuals with HOMA-IR above the p75 had a twice increase in relative risk for cardiovascular events and death. Other indirect evidences also have linked insulin resistance to cardiovascular disease in normal individuals. HOMA-IR has been associated to increased heart rate in healthy sedentary males [16], while glycemia measured by A1C in the non-diabetic range has been shown to correlate with HOMA-IR, being independently related to sub-clinical coronary artery disease [17].

It is intriguing that, in the present study, Matsuda, Stumvoll ISI and OGIS indexes, which are isulin-sensitivity tests based in glycemia and insulinemia after an OGTT, were not associated with CAD. Recently, in a study by FızeI’ova [18], Matsuda index was identified as an important marker that could predict the incidence of type 2 diabetes and CVD events in non-diabetic and newly diagnosed diabetic patients (HR 1.14, p 0.021) [18]. We could partially explain the lack of effect of these insulin sensitivity tests in our study because T2DM were intentionally excluded which substantially decreased the range of 2 h glucose after OGTT results to values below 200 mg/dL. Thus, the smaller range of 2 h glucose values may have decreased the power to detect differences.

An important strenght of this study was the use of the “anatomic burden score” of CAD, described by Mancini et al. [8], which is an independent predictor of death, myocardial infarction and non-ST elevation acute coronary syndrome. There are evidences from recent studies demonstrating that minor degrees of coronary stenosis such as 20 % may be predictive of long-term mortality, when compared to the absence of any epicardial coronary stenosis. Maddox et al., analyzed 37,674 coronary angiographies with different levels of coronary artery disease and identified a significantly higher risk of myocardial infarction in patients with coronary stenosis of just above 20 %. [19]. The coronary artery disease Burden score (CAD Burden), by this way, is of increased clinical relevance [8] because it considers patients with more than 50 % stenosis in which at least one coronary stenosis is necessary to define CAD. Although we have not studied the occurrence of clinical cardiovascular outcomes, the anatomic burden score is considered a strong surrogate for CAD events and, therefore, useful as a reference. Another important strength of this study was the highly homogeneous sample of individuals. This allowed conclusions to the impact of insulin resistance in a non-obese population with near-normal fasting plasma glucose.

The limitations of the present study were that, as a cross-sectional study, no cause-and-effect can be concluded as it may suffer impact of covariates that were not measured in the study. However, the aim of our study was mainly to describe the association between insulin resistance and CAD, which is well known to be associated with adverse cardiac outcomes.

We can conclude that, in patients without diabetes or obesity in whom a coronary angiography study is indicated, a single determination of HOMA-IR above 4.21 indicates increased risk for clinical significant coronary disease. The same association is not seen when using insulin sensitivity indexes such as Matsuda, Stunvoll-ISI or OGIS. Therefore, these findings support the need for further longitudinal research using HOMA-IR as a predictor of cardiovascular disease.
